# Renal metabolic defects in patients with recurrent unilateral Nephro- and ureterolithiasis: insights from individual renal unit sampling

**DOI:** 10.1007/s00240-026-01939-w

**Published:** 2026-01-20

**Authors:** Kevin Stritt, Matteo Ortolini, François Crettenand, Gwaenaelle Aellen, Olivier Bonny, Daniel G. Fuster, Beat Roth

**Affiliations:** 1 Department of Urology , University Hospital Lausanne (CHUV) University of Lausanne , Lausanne, Switzerland; 2 Department of Internal Medicine , Hôpital Fribourgeois (HFR) , Fribourg, Switzerland; 3 Service of Nephrology , Hôpital Fribourgeois (HFR) , Fribourg, Switzerland; 4 Department of Nephrology and Hypertension, Inselspital , Bern University Hospital University of Bern , Bern, Switzerland; 5 Department of Urology, Inselspital , Bern University Hospital University of Bern , Bern, Switzerland

**Keywords:** Nephrolithiasis, Metabolic evaluation, Side difference

## Abstract

Urolithiasis is a highly prevalent condition influenced by environmental, dietary, and genetic factors. Recurrent unilateral stone formation raises the question of whether localized renal metabolic abnormalities contribute to lithogenesis. Conventional 24-hour urine analyses provide a global assessment of urinary solutes and may overlook kidney-specific differences. The objective of this study was to determine whether distinct urinary chemistry differences exist between stone-bearing and contralateral kidneys in patients with recurrent unilateral urolithiasis. This monocentric prospective study was conducted at the Department of Urology, Lausanne University Hospital (Switzerland). Adult patients undergoing elective ureteroscopy (URS) for recurrent unilateral urolithiasis were enrolled. Urine was collected separately from each kidney by selective ureteral catheterization, and samples were analyzed for urinary lithogenic parameters. Paired comparisons were performed using repeated measures ANOVA. Fifteen patients were included (median age 50 year; 67% male; median BMI 24.2 kg/m²), each with a median of three prior stone episodes. Surgical approaches included rigid (47%), flexible (27%), and combined (27%) URS, with a median operative time of 40 min. Calcium oxalate was the predominant stone type (60%), followed by mixed calcium oxalate–phosphate (27%) and uric acid stones (13%). No significant differences were found in urinary chemistry between stone-bearing and contralateral kidneys (all *p* > 0.10). Subgroup analyses by stone type suggested trends toward lower potassium excretion in mixed stones and reduced chloride in urate stones, though these were not statistically significant due to small subgroup sizes.Limitations include the small sample size and single-center design. No significant unilateral metabolic asymmetries were detected in urinary solute handling among patients with recurrent unilateral urolithiasis. This finding does not support the presence of clinically relevant kidney-specific metabolic defects in this cohort and supports the continued use of standard 24-hour urine testing as an adequate metabolic evaluation tool in most clinical scenarios. Kidney-specific urine sampling does not appear justified in routine practice based on the present data and should be limited to carefully selected cases or research settings.

## Introduction

Kidney stone disease is a major public health issue with a lifetime risk ranging from 1% to 20%, depending on geographic, climatic, dietary, genetic, and ethnic factors [1–4]. Kidney stones are not only painful and recurrent but also associated with significant morbidity, healthcare costs, and reduced quality of life [4–7]. Although many stones pass spontaneously, recurrence is particularly common among individuals with underlying metabolic abnormalities [8, 9]. 

In patients at high risk for recurrence, current guidelines recommend 24-hour urine collections to assess metabolic contributors [10]. However, this test reflects the combined excretion of both kidneys, an assumption that may not hold in patients presenting with unilateral recurrent disease only.

Emerging evidence suggests that metabolic asymmetry, especially differences in calcium handling or solute excretion between renal units, may contribute to unilateral stone formation [11, 12]. Reports of unilateral hypercalciuria and variations in urinary calcium-to-creatinine ratios in pediatric and adult populations have raised questions about whether systemic evaluations sufficiently capture the complexity of recurrent stone pathophysiology. Addressing this question is clinically relevant because targeted prevention strategies depend on accurate identification of underlying risk factors.

In this pilot study, we directly compared the urinary chemistry of stone-bearing kidneys with contralateral kidneys in patients undergoing surgery for recurrent unilateral stone disease. By focusing on detailed solute analysis, we sought to determine whether localized metabolic abnormalities are detectable and whether they should influence clinical practice.

## Materials and methods

### Study design and population

This was a prospective, single-center study conducted at the Department of Urology, Lausanne University Hospital (CHUV, Switzerland). The study protocol was approved by the local ethics committee (CER-VD), and all participants provided written informed consent before inclusion. All procedures complied with the ethical standards of the institutional and national research committees and the principles of the Declaration of Helsinki (2013 revision).

Between January and June 2025, fifteen adult patients were prospectively enrolled. Eligible participants had ≥ 2 prior episodes of ipsilateral urolithiasis within the previous 10 years, confirmed by imaging or clinical documentation, and were scheduled for elective URS for a new unilateral stone event. Exclusion criteria included contralateral stones, congenital or acquired urinary tract abnormalities, chronic kidney disease (CKD-EPI eGFR < 60 mL/min/1.73 m²), use of medications known to affect urinary solute excretion, or other systemic causes of stone disease (Table 1). All patients had an indwelling ureteral JJ stent in place at the time of surgery, as part of standard preoperative management.

**Table 1:** Inclusion and Exclusion Criteria of the Study Population

Inclusion criteria
Age ≥18 years
≥3 documented episodes of ipsilateral urolithiasis within 10 years
Scheduled for elective endoscopic stone removal
Fluent in French
Signed informed consent
Exclusion criteria
Contralateral urolithiasis (history or CT evidence)
Kidney size asymmetry >1 cm in length
Nephrocalcinosis
Ureteropelvic junction obstruction or significant obstruction
Congenital or acquired urinary tract abnormalities (e.g., CAKUT, dysgenesis, polycystic kidney)
Kidney transplant recipients
Chronic kidney disease (CKD-EPI eGFR <60 mL/min/1.73 m²)
Current use of medications affecting urinary composition (e.g., thiazide diuretics, xanthine oxidase inhibitors, alkalinizing agents, calcitriol, osteoporosis treatments)
Secondary causes of urolithiasis
Inability to consent or lack of decision-making capacity
Pregnant and lactating women were excluded based on standard surgical contraindications

eGFR = estimated glomerular filtration rate; CKD-EPI = Chronic Kidney Disease Epidemiology Collaboration; CAKUT = congenital anomalies of the kidney and urinary tract

## Study procedures

On the day of surgery, all patients underwent routine preoperative laboratory assessment, including complete blood count (CBC), prothrombin time (PT), activated partial thromboplastin time (aPTT), serum electrolytes (sodium and potassium), and serum creatinine. Procedures were performed under general anesthesia. Ureteroscopy was performed with a pre-existing ipsilateral JJ stent in all patients. After ureteral access was achieved, 5 mL of urine was collected separately from each kidney prior to stone extraction using distinct ureteral catheters to avoid cross-contamination. On the contralateral side, minimal manipulation was ensured by catheterizing the ureteral orifice under direct vision only (without fluoroscopy) to prevent trauma or contamination. All urine samples were processed immediately for biochemical analysis.

### Laboratory Analysis

Urine chemistry included quantification of creatinine, sodium, potassium, chloride, calcium, magnesium, phosphate, oxalate, citrate, urea, protein, and pH, along with microscopic cell counts. Stone composition analysis was determined by Fourier-transform infrared spectroscopy (FTIR), in accordance with EAU recommendations.

#### Statistical analysis

All analyses were performed using Stata version 14 (StataCorp, College Station, TX, USA). Continuous variables are expressed as mean ± standard deviation (SD) or median and interquartile range (IQR), and categorical variables as counts and percentages. Paired comparisons between the stone-bearing and contralateral kidney were performed using repeated measures ANOVA. Given the exploratory nature of the study and the paired design of the data, this approach was retained despite the small sample size. Categorical comparisons used the χ² test or Fisher’s exact test where appropriate. To explore potential unilateral metabolic abnormalities, percent differences between kidneys were calculated and categorized into quartiles; values above the third quartile were considered indicative of asymmetry. Subgroup analyses assessed relationships between urinary solute profiles and stone composition. Potential confounders (age, sex, hydration status, diet, medication use, and comorbidities) were considered in exploratory analyses. The limited sample size and the absence of non-parametric testing are acknowledged as methodological limitations. A two-tailed p value < 0.05 was regarded as statistically significant.

## Results

### Patient demographics and clinical data

A total of 15 patients were included in this study. The median age was 50 years (interquartile range [IQR], 18–78), and 67% (*n* = 10) were male. The median body mass index (BMI) was 24.2 kg/m² (IQR, 20.5–31.3). Regarding the American Society of Anesthesiologists (ASA) physical status classification, 27% (*n* = 4) were ASA I, 53% (*n* = 8) were ASA II, and 20% (*n* = 3) were ASA III. Patients had a median of 3.0 previous stone episodes (IQR, 3.0–4.0), and the median number of stones visible on CT at the time of URS was 1.0 (IQR, 1.0–3.0). Laterality of the stones was nearly evenly distributed, with 47% located on the right and 53% on the left. Surgical intervention consisted of URS, with a median operative time of 40.0 min (IQR, 17.0–95.0). Procedures were performed using a rigid ureteroscope in 47% of cases, a flexible ureteroscope in 27%, and a combined approach in 27%. The demographic, stone history and surgical intervention are shown in Table 2.

**Table 2:** Demographic, stone history and surgical intervention at baseline

Characteristic	Total (N=15)
Demographic	
Median age – yr (IQR)	50 (18 – 78)
Sex:	
Male – no (%)	10 (67)
Female – no (%)	5 (33)
Median BMI – kg/m^2^ (IQR)	24.2 (20.5 – 31.3)
ASA score:	
1 – no (%)	4 (27)
2 – no (%)	8 (53)
3 – no (%)	3 (20)
Stone history	
Median number of past stone episode – no (IQR)	3.0 (3.0 – 4.0)
Side of stones:	
Right (%)	7 (47)
Left (%)	8 (53)
Median number of stones – no (IQR)	1.0 (1.0 – 3.0)
Intervention	
Median operative time – no (IQR)	40.0 (17.0 – 95.0)
Type of URS:	
Rigid	7 (47)
Flexible	4 (27)
Rigid and flexible	4 (27)

IQR: interquartile range, BMI: body mass index, ASA score: American Society of Anesthesiologists score, URS: ureteroscopy

### Stone composition

Stone analysis revealed calcium oxalate as the dominant component in 60% of patients, primarily as calcium oxalate monohydrate. Mixed calcium oxalate–phosphate stones accounted for 27%, while uric acid stones represented 13%. These results align with global epidemiological data, underscoring calcium oxalate as the most frequent stone type.

### Urinary chemistry findings

Paired comparisons of urinary solutes between stone-bearing and contralateral kidneys demonstrated no significant differences in urinary concentrations of sodium, potassium, chloride, calcium, magnesium, phosphate, protein, urea, citrate, oxalate, or pH (all *p* > 0.10). Creatinine concentrations were also similar, indicating equal urine flow rates. These findings suggest that systemic metabolic factors, rather than unilateral abnormalities, predominantly influence urinary composition. Notably, the stone-bearing kidney exhibited significantly higher urinary erythrocyte, and leukocyte counts compared to the unaffected side (erythrocytes: mean 1530.6 vs. 358.8 G/l, η² = 0.607, *p* < 0.001; leukocytes: mean 24.5 vs. 6.4 G/l, η² = 0.372, *p* = 0.012), likely reflecting local inflammation or trauma (Table 3 and 4).

**Table 3:** Comparison of Urinary Parameters Between Stone-Bearing and Contralateral Kidney (Repeated Measures ANOVA)

Urine Parameter	Kidney with Stone (Mean ± SD)	Kidney without Stone (Mean ± SD)	η²	p-value
Creatinine (µmol/L)	1796.2 ± 1705.3	2377.2 ± 2313.8	0.092	0.254
Sodium (mmol/L)	120.5 ± 17.8	130.2 ± 28.7	0.153	0.134
Potassium (mmol/L)	23.9 ± 9.7	28.3 ± 13.8	0.103	0.226
Chloride (mmol/L)	129.3 ± 17.8	135.6 ± 27.4	0.047	0.421
Calcium (mmol/L)	1.3 ± 0.5	1.4 ± 0.6	0.043	0.442
Magnesium (mmol/L)	1.3 ± 0.5	1.2 ± 0.5	0.014	0.665
Phosphate (mmol/L)	2.4 ± 2.6	2.6 ± 2.5	0.005	0.788
Protein (g/L)	0.4 ± 0.4	0.4 ± 0.7	0.004	0.812
Urea (mmol/L)	43.9 ± 33.7	57.8 ± 43.5	0.141	0.152
Citrate (mg/L)	63.1 ± 63.2	102.7 ± 123.7	0.137	0.158
Oxalate (µmol/L)	42.7 ± 53.9	49.9 ± 47.9	0.029	0.530
pH	6.6 ± 0.7	6.5 ± 0.7	0.178	0.104
Urine Sediment				
Erythrocytes (G/L)	1530.6 ± 611.9	358.8 ± 440.6	0.607	<0.001
Leukocytes (G/L)	24.5 ± 22.7	6.4 ± 7.4	0.372	0.012

Mean values ± standard deviation are shown for each parameter. Repeated measures ANOVA was used to compare solute concentrations and cell counts between renal units. No significant differences were observed in urinary solutes, while the stone-bearing kidney showed significantly elevated erythrocyte and leukocyte counts (p < 0.001 and p = 0.012, respectively), likely reflecting local inflammation or trauma.

### Subgroup analysis by stone type

When stratified by stone composition, certain trends emerged with significant differences for potassium (*p* = 0.046, η² = 0.286) and chloride (*p* = 0.004, η² = 0.210) (Table 4). Patients with mixed stones had the lowest potassium levels, while those with urate stones exhibited the lowest chloride concentrations. Although not statistically significant, urinary calcium was lowest in urate stone formers (mean 0.5 mmol/L) compared to calcium oxalate (1.5 mmol/L) and mixed stones (1.2 mmol/L), with a relatively large effect size (η² = 0.431). Similary, urinary pH tended to be lower in patients with urate stones compared to patients with calcium stones. Phosphate levels were markedly elevated in the urate group (mean 7.0 mmol/L), though this did not reach significance (*p* = 0.187). No relevant differences were seen for citrate, oxalate, protein, or pH. These results indicate that underlying metabolic differences may influence stone composition, although the limited subgroup sizes—especially for urate and mixed stones—constrain statistical robustness and generalizability.

**Table 4:** Comparison of Urinary Parameters According to Stone Composition

Parameter	Calcium Oxalate (N=10)	Mixed calcium (N=3)	Urate (N=2)	η²	p-value
Creatinine (µmol/l)	1997.2 ± 1868.9	563.0 ± 334.6	2641.0 ± 1630.6	0.157	0.202
Na (mmol/l)	122.0 ± 21.3	123.0 ± 4.4	109.5 ± 4.9	0.064	0.097
K (mmol/l)	26.9 ± 10.1	14.0 ± 2.6	23.5 ± 3.5	0.286	0.046
Cl (mmol/l)	134.3 ± 19.7	124.1 ± 3.5	112.0 ± 1.4	0.210	0.004
Ca (mmol/l)	1.5 ± 0.4	1.2 ± 0.4	0.5 ± 0.4	0.431	0.137
Mg (mmol/l)	1.4 ± 0.5	0.8 ± 0.1	1.5 ± 0.3	0.294	0.064
P (mmol/l)	1.9 ± 1.8	0.7 ± 0.3	7.0 ± 3.9	0.542	0.187
Protein (g/l)	0.3 ± 0.9	0.6 ± 0.6	0.7 ± 0.5	0.152	0.667
Urea (mmol/l)	45.5 ± 32.8	18.0 ± 11.5	75.1 ± 44.5	0.250	0.22
Citrate (mg/l)	66.4 ± 56.5	20.0 ± 0.0	111.5 ± 129.4	0.186	0.292
Oxalate (µmol/l)	48.7 ± 61.7	10.0 ± 0.0	61.5 ± 47.4	0.105	0.515
pH	6.7 ± 0.7	6.8 ± 0.3	6.0 ± 0.7	0.149	0.38

## Discussion

This prospective study evaluated urinary chemistry in patients with recurrent unilateral nephrolithiasis using paired intraoperative renal unit sampling. Contrary to the hypothesis of significant unilateral metabolic abnormalities, no consistent differences in solute excretion were observed between stone-bearing and contralateral kidneys. These findings indicate that no clinically relevant kidney-specific metabolic asymmetry was detectable in this cohort. On this basis, and in the absence of detectable unilateral differences, our results are consistent with the continued use of systemic metabolic evaluation, such as 24-hour urine analysis, in most clinical scenarios, although a direct comparison with 24-hour urine collections was not performed in this study.

Stone composition analysis revealed that calcium oxalate monohydrate was the predominant component, consistent with global epidemiological data identifying calcium oxalate as the most prevalent stone type in both men and women.¹ When urinary biochemical profiles were compared across stone composition groups, statistically significant differences in potassium and chloride concentrations were observed. These variations may reflect distinct underlying pathophysiological mechanisms contributing to stone formation. For instance, lower urinary potassium has been linked to inadequate dietary intake or impaired renal handling, both of which can decrease citrate excretion and increase stone risk [13]. Likewise, altered chloride levels may indicate acid-base disturbances, particularly relevant in the context of uric acid stone formation [14]. Although the small sample size, particularly within the urate and mixed stone groups, limits definitive conclusions, these findings support the notion that specific stone types arise within distinct metabolic milieus [15]. Further studies in larger, well-characterized cohorts are warranted to validate these associations and clarify their clinical implications for individualized prevention strategies.

Our findings also align with previous reports suggesting that, although stones often form unilaterally, the underlying lithogenic risk factors are frequently systemic rather than confined to a single kidney [11, 12]. The absence of significant unilateral metabolic differences in our cohort suggests that routine intraoperative sampling of both kidneys may offer limited additional diagnostic value. This interpretation is based on the lack of detectable inter-renal differences rather than on a direct comparison with pooled or bladder urine samples. This consideration is particularly pertinent given the added procedural time, instrumentation, and potential risk associated with bilateral sampling. Nonetheless, in selected patients—especially those with recurrent ipsilateral stone formation despite unremarkable 24-hour urine analyses—individualized renal unit evaluation may still be clinically justified [16, 17]. In such cases, targeted assessment could reveal subtle tubular defects or localized excretory discrepancies not captured by standard global urine collection methods, thereby informing more tailored preventive approaches.

Urine sediment analysis demonstrated significantly elevated levels of erythrocytes and leukocytes on the side of the stone-bearing kidney. These findings are most likely attributable to procedural factors, including ureteral instrumentation and the presence of an indwelling JJ stent in all patients, rather than reflecting intrinsic metabolic differences. JJ stents has been associated with increased urothelial irritation and local inflammatory responses [18]. Accordingly, erythrocyte and leukocyte counts were not interpreted as mechanistic contributors to stone formation in this study [19]. Notably, no calcium oxalate crystals were observed in the sediment. This absence should be interpreted cautiously, as intraoperative irrigation causing dilution or to the limited urine volume collected during the procedure, both of which may reduce the sensitivity of crystal detection [20]. 

This study has several limitations. The relatively small sample size limits statistical power and the generalizability of the findings, particularly in subgroup analyses based on stone composition. Given the limited sample size, non-parametric statistical testing may have been preferable; however, the exploratory and paired design of the study led us to retain parametric analyses, which should be interpreted with caution. Moreover, perioperative factors—including variations in hydration status, anesthesia-related physiological changes, and the presence of indwelling ureteral JJ stents in all patients at the time of urine collection—may have influenced urinary composition, potentially confounding the assessment of true metabolic asymmetry. Previous studies have also noted that stent-related urothelial irritation can alter urinary parameters, particularly leukocyturia and hematuria [21]. Exclusion of acquired urinary tract abnormalities was based on clinical history and standard imaging; subtle regional renal scarring or functional asymmetry may therefore not have been fully excluded. Functional imaging such as renal scintigraphy was not performed, as it was not part of routine preoperative assessment in this exploratory study. Reliable urine volume measurement was not feasible in the intraoperative setting due to irrigation and short sampling duration, and urinary parameters could therefore not be systematically normalized to volume or creatinine. As a result, this exploratory analysis should be considered hypothesis-generating rather than definitive. Future research involving larger, multicenter cohorts and standardized perioperative protocols is warranted to better characterize potential unilateral renal metabolic differences. Such investigations may also help to identify specific patient subgroups—such as those with persistent ipsilateral recurrence despite unremarkable 24-hour urine profiles—who could benefit from targeted, renal unit–specific metabolic evaluation [11, 12]. 

## Data Availability

No datasets were generated or analysed during the current study.
